# Peer Outreach Work as Economic Activity: Implications for HIV Prevention Interventions among Female Sex Workers

**DOI:** 10.1371/journal.pone.0119729

**Published:** 2015-03-16

**Authors:** Annie George, Kim M. Blankenship

**Affiliations:** 1 Independent Research Consultant, 206 Sun City B, NIBM Road, Pune, India; 2 Department of Sociology and Center on Health, Risk and Society, American University, Washington, D.C., United States of America; UNC Project-China, CHINA

## Abstract

Female sex workers (FSWs) who work as peer outreach workers in HIV prevention programs are drawn from poor socio-economic groups and consider outreach work, among other things, as an economic activity. Yet, while successful HIV prevention outcomes by such programs are attributed in part to the work of peers who have dense relations with FSW communities, there is scant discussion of the economic implications for FSWs of their work as peers. Using observational data obtained from an HIV prevention intervention for FSWs in south India, we examined the economic benefits and costs to peers of doing outreach work and their implications for sex workers’ economic security. We found that peers considered their payment incommensurate with their workload, experienced long delays receiving compensation, and at times had to advance money from their pockets to do their assigned peer outreach work. For the intervention these conditions resulted in peer attrition and difficulties in recruitment of new peer workers. We discuss the implications of these findings for uptake of services, and the possibility of reaching desired HIV outcomes. Inadequate and irregular compensation to peers and inadequate budgetary outlays to perform their community-based outreach work could weaken peers’ relationships with FSW community members, undermine the effectiveness of peer-mediated HIV prevention programs and invalidate arguments for the use of peers.

## Introduction

Globally, female sex workers (FSWs) are at higher risk of HIV than the general population [1]. In India, too, when in 2006 the HIV prevalence among the general adult population was estimated to be 0.36 percent, it was estimated at 5.38 percent among FSWs [2]. HIV prevention interventions targeted at FSWs often use them as community health workers (CHWs), also called peer educators (PEs), to achieve high coverage, impact HIV outcomes, and promote FSW empowerment and ownership of HIV prevention programs [3–7]. A systematic review of the role and impact of community health workers (CHWs) in HIV interventions in sub-Saharan Africa [8] found that CHWs were seen as a bridge between the community and health facilities [9], and that programs that used CHWs reported increasing uptake of HIV services [10, 11]. Evidence from HIV prevention interventions in India that use peers achieve such desired outcomes among FSWs as increased reported use of condoms with clients, reduced incidence of STIs [12, 13], and reduced incidence of reported violence [14]. Evidence also suggests that PEs’ work to mobilize FSWs in terms of strengthening collective solidarity [15], forming and strengthening community-based organizations (CBOs) [16–19], advocating with police and other stakeholders [16, 20] and campaigning for social entitlements [20] may lead to desired HIV prevention outcomes. This evidence suggests that involving FSWs as PEs or CHWs is critical for effective programmatic responses to HIV prevention among FSWs. Yet, studies of CHWs in HIV prevention report high rates of attrition [8].

Attrition of CHWs is attributed in part to the economics of work as CHWs [8, 21], and related to such monetary factors as inadequate and irregular pay [22–24], loss of other economic opportunities, and better employment in other fields [25]. For example, CHWs in Kolkata who received a monthly honorarium, which they considered inadequate, were reported to be ambivalent about their volunteer status and demanded the NGO for which they ‘volunteered’ pay them a ‘salary’ equivalent to their ‘work’ [26]. In Bangladesh, women who depended on income as CHWs were reportedly more actively involved in their outreach work than those who did not depend on this source of income [27].

The use of members of low-income communities as CHWs by NGOs supported by international donors has raised critical commentary on issues of program sustainability and ethics [28–30] and the need to consider the value and fair compensation of the labor of CHWs [21, 31]. A global review of CHW programming noted that CHWs are poor people who need an income and that while they are expected to spend only a small amount of time on their health-related duties and the rest of their time on earning a livelihood, in reality their work often requires full-time performance ([32] p.27). Recognizing the need for effective programming and the retention of community members in providing outreach services in public health programs, the World Health Organization (WHO) recommended that CHWs be paid a fair and regular wage to secure not only their commitment to the program but also their livelihood [33].

CHWs receive monetary compensation and are held accountable and supervised like paid employees [21], yet their work does not provide secure income, worker benefits, social protection, or legal status. Although their work conditions are similar to those of workers in the informal economy [34], CHWs are perhaps considered volunteers because historically and contemporarily interventions globally depend on them to volunteer their time and labor to provide community-level services [31, 35–39]. Even when CHWs receive monetary and some social benefits for their work, it is not regulated or protected under labor legislation or social protections. This is so even as the WHO calls on countries to consider shifting tasks from highly skilled health workers to workers with shorter training, including CHWs, to achieve the goal of universal access to HIV services [33]. At the same time, the WHO also acknowledges that to be sustainable, health services cannot be provided by volunteers and that all workers, including CHWs “…should receive adequate wages and/or other appropriate and commensurate incentives” ([33] p.4). Clearly then, while there is empirical evidence of the *benefits to HIV prevention* efforts from engaging the work of PEs, there is scant empirical evidence of the *economic implications for FSWs* of working as PEs. This paper attempts to fill this gap.

HIV prevention programming for FSWs in India has been supported by both the Government of India’s National AIDS Control Program (NACP) and the Bill & Melinda Gates Foundation’s (BMGF) Avahan India AIDS Initiative [40, 31]. Both interventions used FSWs as PEs to work with non-government organizations (NGOs) and/ or with community-based organizations (CBOs) comprised solely of FSWs or former FSWS. Each type of organization was contracted to, among other things, use community mobilization strategies with local FSWs to reach desired HIV-prevention outcomes. Avahan-supported peers were expected to work four or five hours a day, six days a week and make systematic and regular contact with around 50 FSWs. They provided FSWs with a range of outreach services including information and counseling about HIV prevention, condoms, and referrals to STI clinics and HIV testing centers. Additionally, PEs had to create an enabling environment for FSWs by mobilizing them into collectives (CBOs), advocating for FSWs’ needs with key stakeholders, responding to crises faced by FSWs, and providing them legal rights education. They received a monthly honorarium equivalent to US$ 20-$30 to recognize that the time they spent as PEs meant a loss of income from their regular work, and to act as ‘a kind of positive acknowledgment of peers' work [that] helps to retain them in the program’ ([41] p.13). For a similar work profile, NACP-supported PEs received between US$ 25-$30 per month to compensate for their time and travel-related expenses [40]. How does this compare with money they earn as sex workers, or from other work opportunities they may have had? In general, FSWs’ actual and potential earnings from sex work were greater than earnings from work as PEs, which involved time away from sex work and a potential loss of earnings that was not compensated from their work as PEs.

Studies have found that approximately half of sex workers in India have another means of livelihood in addition to sex work; many of them work as unskilled laborers in the agricultural and non-agricultural sectors [42, 43]. In 2009–2010, the national average *daily* wage rates for unskilled women laborers in the agricultural and non-agricultural sectors was, respectively, between Rs 47–87 (USD 0.85–1.60) and Rs 73—Rs 82 (USD 1.30–1.50) [44]. The average *monthly* earning of FSWs was reported to be Rs. 4621 ± Rs.3098 (USD 84 ± USD 56) [45]. FSWs who worked at other occupations earned between Rs. 500–1000 per month from that occupation, whereas the modal and median incomes in sex work were respectively Rs. 1000–3000 and Rs. 3000–5000 [43]. When these findings are considered alongside other findings that the majority of FSWs support two or more dependents [46], and are indebted to moneylenders [47], and those FSWs who migrated away from their place of origin for sex work did so to repay debts, among other reasons [48, 49], it raises questions about the costs to FSWs of working as PEs, and also the efforts made by HIV prevention interventions, in terms of monetary policies, to retain PEs.

The two-fold purpose of this paper is to provide empirical evidence of the economic implications for FSWs of being PEs, and through this, to revisit the issue of the policy and programmatic considerations to reduce attrition of CHWs in HIV prevention programming and thereby positively affect desired HIV prevention outcomes. We base our arguments on ethnographic data on the implementation of a peer-mediated HIV prevention intervention in Andhra Pradesh, India that was initially funded and managed by Avahan-supported NGOs and then transitioned to the state-run Andhra Pradesh State AIDS Control Society [50, 51].

## Methods

This paper is based on data drawn from Project Parivartan, a multi-methods study supported by the BMGF to analyze the implementation and impact of peer-mediated community mobilization interventions to reduce vulnerability and HIV risk among FSWs in select districts of Andhra Pradesh state in southern India. From October 2004 through June 2007, Project Parivartan’s researchers observed the working of one NGO that provided HIV prevention services in the areas around Rajahmundry town in East Godavari District. From 2008 through 2012, Project Parivartan expanded its research to six NGO- and one CBO-managed HIV prevention interventions in and around Nellore and Guntur towns in the eponymously named districts in Andhra Pradesh. Data for this paper are based on field research conducted for 12 months starting from January 2012 when, at all three research sites, the funding and management of the HIV intervention was being transitioned from Avahan to the state government’s Andhra Pradesh State AIDS Control Society. In 2012, the ratio of peer outreach worker to FSW at all interventions across the state was set at 1:50; interventions at the three sites reported a total of 233 peer outreach workers who provided services to 13720 FSWs.

The field research was coordinated by two teams, one in India and the other in Washington DC. In India, a team of three ethnographers visited Nellore and Guntur three times each and Rajahmundry twice; at each site they spent around 10 days per visit. They observed but did not participate in staff meetings and at the sidelines they met NGO and APSACS staff. They followed peer outreach workers on their outreach work and in the course of this field work, spoke with outreach workers, FSWs, and facility personnel. They also spent time at the NGO and CBO offices where they spoke to peer outreach workers, intervention managers and workers and CBO leaders. The conversations were unstructured, although researchers had a list of broad topics to be covered with different participants; the ethnographers’ goal was to gain information that would illuminate the process of transition of the intervention from the BMGF to APSACS, and the challenges faced in the process and how these were addressed.

For this paper we draw from 8 records of research team meetings, 14 observations of peer meetings, 14 observations of intervention staff meetings, and 50 observations of peer workers at the NGO office and on their outreach activities at government health facilities and in areas where FSWs worked and lived. During these observations researchers held 60 brief discussions with FSWs working in a range of different sex work settings, of various marital and family situations and ages, on their economic situation, income earned from various sources and their routine household expenses. The first author was involved in the direct supervision of data collection. Tables 1 and 2 provide details respectively of the activities observed and category of participants with whom researchers talked.

**Table 1:** Activities observed to understand the work of peer outreach workers in an HIV-prevention intervention for FSWs.

Activity observed (number of observations)	Observation points
Peer outreach worker meetings (14)	Topics on agenda that were discussed and not discussed, response of intervention staff to issues raised by peers.
Intervention staff meetings (14)	
Peer workers at the NGO/CBO office, doing outreach work at clinics, sex worker hot spots & places where FSWs lived (50)	Payment for outreach work, expenses relating to outreach work, modes of monitoring work done, record keeping of work done, challenges & benefits relating to work as peers, sources of income, loans taken, sources of loans, savings.

**Table 2:** Participants with whom informal conversations were held to understand costs and benefits to FSW of working as peer outreach workers.

Category of participant	Topics covered
FSW	Family size & dependents, sources of income, types of expenses, loans taken, sources of loans, savings.
Peer outreach worker	Benefits & costs of doing peer outreach work, challenges to meeting targets, payment delays, costs of transporting FSWs to clinics, process of supervision & monitoring peer outreach work.
Supervisors of peer outreach workers	Challenges of monitoring peer outreach work, reaching targets, dealing with payment delays.
Clinic staff (at government & intervention-run clinics)	Challenges peers face in bringing FSWs for routine preventive STI checks.
Project Manager	Recruitment & retention of peer outreach workers, payment for PEs, monitoring & supervising peer work, challenges faced, setting & achieving work targets, interactions with peer workers & other intervention staff.

The study received ethical approval from American University’s Institutional Review Board and the Institutional Review Board of Y.R.G. Centre for AIDS Research Education (YRGCARE), a research institution in India. These boards approved observation of a wide variety of key activities relating to ongoing activities associated with project management, development of new activities, maintenance of linkages with outside organizations and community groups, negotiations with authorities, and maintenance of community involvement in the intervention. Prior to starting the research, ethnographers met the staff of all NGOs and CBOs that were involved in the intervention and explained the research objectives and methods, sought verbal consent for the researchers’ presence at meetings and other intervention activities, and clarified the processes put in place to keep confidential the identities of all persons with whom researchers interacted. The two institutional boards approved this process of obtaining consent since the focus of the ethnographic research was less on observing individuals as individuals and more on understanding the broader context of sex work and the general operation of the intervention.

The India-based ethnographers recorded their notes on site and expanded them immediately after each visit. Each 10-day field immersion was followed by a day-long debriefing meeting with other India team members, including the first author; the discussions served as a preliminary analysis of the field data and a way to identify areas for further investigation at the next site visit. A detailed write-up of these team meetings were shared with team members at American University, including the second author, who provided inputs for further areas of information gathering.

Preliminary data analysis was on-going with the field research. Once field research ended, we used thematic content analysis to analyze the data [52]. The first author read the entire data set several times to obtain a sense of the whole data set, and then identified emerging themes and coded the data using NVivo 10. All data related to peers and monetary considerations were identified, coded and sorted into distinct sub- sets. Through several rounds of close reading of the coded data, similar content was sorted into the following themes: (1) the economic situation of PEs and other FSWs, (2) work as PE offers potential to also earn money through brokering, (3) low honorarium for PE work does not compensate for loss of income from sex work and (4) expenses incurred to work as PEs, including costs of travel, mobilizing FSWs and paying for the distribution of socially marketed condoms. At research meetings, both authors periodically discussed emerging interconnections between codes and themes. Findings are presented under these themes.

## Findings

PEs, like most of their FSW peers, reported some degree of economic vulnerability. Outreach work provided PEs a monthly honorarium from the intervention, as well as a commission from brokers and clients for referring FSWs under their charge to them. Yet, PEs found themselves in an economic bind. The low honorarium for PE outreach did not compensate for loss of income from sex work that resulted from the time spent on PE work rather than sex work. Further, to achieve their targets, PEs had to pay a number of different and unanticipated out-of-pocket expenses towards travel costs, mobilizing FSWs and distribution of socially marketed condoms. Non-achievement of targets for outreach work could result in dismissal from the position. These issues are discussed next.

### The economic situation of PEs and other FSWs

PEs and other FSWs were economically insecure. Discussions with 60 FSWs at the three sites, seven of whom were peer outreach workers, revealed that the women had, on average, at least two dependents; 39 women supported children aged less than 18 years. Twenty three women did some other work besides sex work, such as daily wage labor in agro-industries, peer outreach work, agricultural labor and running small shops. Twenty four women said that for want of money they had missed a meal in the past week. Forty six women lived in rented houses, and of these 18 women said they were unable to pay the preceding month’s house rent. Fifty six women had outstanding loans; of these 49 women said their loans were taken in the six months preceding the conversation with the researcher. Most borrowed from private money lenders who charged high interest rates repayable in daily, weekly or monthly installments. For instance, a typical interest rate for weekly repayment was Rs 3 per Rs 1,000 borrowed. Most women’s loans ranged between Rs 2,000 and Rs 20,000. Fourteen women said they had saved money in the past six months. Overall, most FSWs were in debt, had few savings, supported dependents and were frequently unable to pay for food or housing.

### Peer work offers potential to earn money through brokering

The economic benefits of work as PE were twofold: a monthly honorarium of Rs 1,500 and Rs 200 to compensate for outreach-related travel; and commission from brokers and clients for referring FSWs under their charge. From the peers’ perspective their work as PE and broker could be complementary and remunerative. Women selected as PEs were active sex workers and well-networked leaders of the FSW community who considered the position of peer as a way to bolster their leadership standing, not only by linking FSWs to the services provided by the intervention but also by linking FSWs to brokers and clients.

Each peer was allocated 50–60 FSWs to whom she was required to provide outreach services; she also had easy access to them whenever she had clients who wanted sexual services. As a broker linking FSWs to clients, the peer earned a commission and the loyalty of FSWs to whom she referred clients. Referrals, PEs claimed, increased their legitimacy among FSWs who, consequently, were more likely also to comply with such HIV prevention advice as regular STI checks and condom use with clients. Our data indicate that some PEs in Nellore and Rajahmundry and all peers in Guntur worked as brokers for brothels and clients; for each referral brokers typically earned 30 to 50 percent of the charges paid by clients to FSWs. Occasionally they earned even more. A peer at Rajahmundry said that brokers charged wealthy clients tens of thousands of rupees, a portion of which she received as commission, and a peer from Guntur revealed that she earned around Rs 20,000 per month from sex work and commissions from referrals.

Despite the potential earnings from peer outreach work, PEs expressed reservations about the economic benefits of such work. By definition, PEs were meant to be practicing FSWs with active client networks who spent only part of their time on outreach for HIV prevention. In reality peers said they spent much time in outreach and documentation of their work; without such documentation, they would be considered ineffective by the NGO/CBO. They worked despite compensation from the NGO/CBO they considered to be inadequate and that was frequently delayed. These issues are discussed next.

### Low honorarium for peer outreach does not compensate for loss of income from sex work

Across the three sites, at staff meetings, PEs informed Project Managers that, relative to their work load, the honorarium was low. Reflecting the sentiments of her colleagues, a peer said,

“We have to cover 60 FSWs…we need to submit many reports…we are doing so many things for a low salary…we are paid only Rs.1700….in addition they are cutting Rs 200 from our salaries for condom social marketing and if we couldn’t reach clinic targets they threaten that salaries will not be given.”

This excerpt highlights peers’ view that remuneration was inadequate and yet they were threatened with economic sanctions for poor performance, the latter measured as not reaching their targets. Other project staff concurred with peers’ views about their low payment. A Project Manager said given this low level of compensation, ‘How can a peer outreach worker of that age [18 to 35 years, the peak years when FSWs can earn] concentrate to save the lives of 60 FSWs leaving her own [sex work] business?’ This respondent’s remarks suggests that, in the context of delayed payments, it was not possible for PEs to meet their work targets, and that when payments were low and delayed, it was unrealistic of interventions to expect peers not to focus on earning money from sex work. Interventions deliberately selected well-networked FSWs, yet this very criterion appeared to affect their work as peers. At several meetings across the three sites, their supervisors complained that PEs were not present and available when they made supervisory site visits. Peers said, in explanation, that they needed to actively nurture their sex work networks because FSWs’ compliance with HIV prevention messages and use of services was motivated in part by the expectation of referrals for sex work business. Confirming this view, the Project Manager at Nellore said that not many FSWs were willing to work as peers, and that those who agreed did so not only for the honorarium, but also because it allowed them to cultivate their sex work business as brokers and sex workers. Yet, FSWs’ condition that their use of HIV prevention outreach services was contingent on client referrals was a source of pressure on peers. A peer said,

“…many FSWs insist that we provide them business. They say ‘We can get or buy condoms, visit government clinic for tests, why do we need to attend the NGO/CBO meetings?’ and [they] insist upon [us] referring clients. Then only will FSWs be willing to attend our meetings…We visit DIC [drop in center] not only to take rest … but even to fix a party [client for a FSW].”

This quote shows the interconnections between work as broker and as peer and indicates that PEs depended as much on FSWs to achieve their peer outreach targets as FSWs depended on peers for client referrals. Some peers said that they relinquished referral-based commissions from FSWs so as to retain their assigned FSWs, and achieve their monthly targets.

Peers said that they did not receive honorariums and travel money for several months consecutively, which negatively impacted their work. Periodically, at all sites PEs reported at meetings that for want of travel money they did not reach out to FSWs and could not meet targets. Sporadic provision of outreach services meant that when peers visited FSWs, they had to spend more time with them re-establishing their credibility as peer outreach workers. Yet peers feared that non-achievement of targets would affect their job tenure. When payments were delayed, some peers did not do outreach work whereas others took loans to cover the costs of doing the work. Talking about the effect of payment delays on peers, a clinic staff told researchers that despite these delays, PEs

“…managed to bring the FSWs for regular medical checks and voluntary HIV tests because they borrowed money to cover outreach and transport costs… after the salary was released, they cleared the debts…”

Even when they met their assigned targets, at some sites PEs were paid their honorarium only after their supervisor certified that the peer had documented the assigned work to her satisfaction. This sometimes resulted in further delays in payment. Peers’ insecurity of tenure was strengthened by a then recent NACP policy that peers could be no older than 35 years and their tenure no longer than 18 months. Not all sites implemented this policy fully; yet many peers across the three sites, particularly those 35 years and older, were retrenched and interventions were unable to recruit replacements partly because the remuneration did not attract younger FSWs. Project Managers at all sites said that at various times during 2012, the budgeted positions of PEs remained unfilled. Speaking of the low payment for peers as a constraint to recruitment, a staff member remarked that all peers and potential peers were active sex workers who could earn Rs 1500 from two clients. This rhetorical statement notwithstanding, the informant’s point was that peers could earn more money from sex work than from work as outreach workers. Despite wavering economic returns, PEs said they remained on the job anticipating that the government would regularize their positions, following which they could expect a regular income.

### Expenses incurred to work as PEs

The economic benefits of work as peer educators were offset by the money peers spent from their pockets on travel and community mobilization costs and paying for socially marketed condoms. These expenses and the situations leading to them are discussed next.

Travel costs

PEs incurred travel costs to reach their assigned outreach site and transport peers to clinics, often several times, if medical personnel or testing kits were not available at the first visit.

Although the intervention required peers to reside in the area where they did outreach, some peers with family ties in the rural areas lived in towns in order to benefit from the larger pool of potential clients and avail the urban cover of anonymity. For instance, at a staff meeting, a staff person complained that four of the 17 peers she supervised were rarely available at their designated work sites in the villages. Explaining why they were not always available at their designated sites, peers said that their work timings as peers was flexible and that they tried to economize on travel costs between the town where they lived for sex work opportunities and their designated site for peer outreach work.

Peers also incurred travel costs to meet their assigned FSWs and transport them to clinics. During the period of our field research, each peer had to refer 60 percent of her designated FSWs to an NGO clinic and the remainder to government health centers, both of which were located at the main city of each district; to reach them on public transport from outlying villages typically took more than an hour. At several meetings to review peers’ work, when the discussion centered on the non-achievement of targets, peers informed staff that the Rs 200 travel allowance was far less than their actual travel costs, which affected their outreach work and targets. Speaking about this situation a peer said,

“When girls do not have business we pay the auto fare and take them to Government clinic for tests. Testing kits were not available at the government health center last month. So this month we have to complete those also.”

This excerpt indicates that peers’ transport costs were affected by variability of FSW earnings, non-availability of test kits at clinics which necessitated repeated visits to motivate FSWs to return to the clinic, and pressure to achieve targets.

Costs of ‘mobilizing’ FSWs

To meet their targets of mobilizing FSWs, PEs were exhorted to pay CBO membership fees of FSWs assigned to their charge; they paid partial costs of monthly rent of a space where FSWs could meet. Furthermore, PEs bore or were being pressured to bear costs related to actions that would help PEs mobilize FSWs. For instance, at Nellore and Rajahmundry peers reported at monthly meetings that they were unable to meet the target for collecting CBO membership dues from FSWs under their charge. Although during the time of our field research PEs were not required to pay these membership dues, at monthly meetings intervention staff repeatedly urged them to bear this cost, arguing that it was something that they could ‘contribute’ to the FSW community. This, despite the situation of peers at all sites reporting non-receipt of monthly honoraria for several months at a stretch. In another instance of mobilization-related expenses, peers at Nellore reported that in the transition phase the intervention cut back on full payment of rent of space for a Drop-in-Center, where FSWs rested when not meeting clients and where peers met their FSWs. To make up the shortfall, peers were each contributing Rs 75 per month from their pockets. These instances indicate that to secure their positions as PEs, they sometimes had to bear the costs of mobilizing FSWs.

Paying for socially marketed condoms

PEs bore costs associated with meeting the target for selling socially marketed condoms. Socially marketing of condoms is a strategy to distribute condoms to difficult to reach populations. Interventions provide PEs with subsidized condoms which they in turn sell at an affordable price to those of their FSW peers and clients who can afford them. PEs were rewarded financially from margins on their sales. Socially marketed condoms were expected to complement free distribution and commercially sold condoms. During the time of our observations, the intervention’s policy relating to the distribution of male condoms by peers in the first year of transition was that 80 percent would be given free and 20 percent would be ‘socially marketed’. Over time, the PEs’ proportion of socially marketed condoms was to increase and freely distributed condoms decrease. In practice, this meant that peers bought the socially marketed condoms from the intervention at below-market rates and sold them to FSWs and others at market rates; the profit was shared between the peer and the local CBO. Intervention staff was responsible for collecting the CBO share from peers and handing it to the CBO. Peers at Nellore and Rajahmundry reported at weekly review meetings that they were unable to meet their targets for socially marketed condoms. The Project Manager told researchers his supervisor insisted that he recover the CBO’s share of profit from the sale of socially marketed condoms; he solved the problem by deducting in advance from their monthly honorarium the amount PEs would have to give the CBO for the sale of the socially marketed condoms. This way, he could report to his supervisor that the socially marketed condoms were sold and the money recovered. A similar situation existed at another site where a peer said that without their consent, and regardless of the actual number of condoms they sold, the Project Manager deducted in advance from peers’ monthly honorarium the money expected from the targeted sale of socially marketed condoms, which was due to the CBO. These instances indicate that PEs had to absorb both the costs and the pressure faced by intervention staff to meet targets for the sale of socially marketed condoms.

## Discussion

Our findings demonstrate that, among other things, peer outreach work is an economic activity for FSWs and they reiterate the importance of monetary considerations for poor women who are drawn to work as peer outreach workers. In this regard, they echo Lehmann’s (2009) observation regarding CHWs (of which PEs are a type), namely, that although in many programs, CHWs are expected to spend only a small amount of time on their health-related duties and the rest of their time on earning a livelihood, in reality the work of a CHW often requires full-time performance. Their compensation, typically paid as an honorarium, often is not adequate to the work that they do. Similarly, in the case of the HIV prevention intervention among FSW that we observed in India, despite adopting a peer-mediated strategy, the intervention neither gave adequate thought to the economic conditions of PEs and what doing outreach work would entail in economic terms for them nor provided adequate budgetary allocations in a timely manner to help retain peers. Our findings suggest that instead of mitigating the economic vulnerability of peers, presumably by paying them an honorarium in recognition of the work that they do, interventions contribute to their economic vulnerability. In turn, they raise questions about how peers can be retained so as to maintain program effectiveness and outcomes. Lessons from systematic review of work with CHWs in other public health programs may offer insights for peer-mediated HIV prevention programs for FSWs.

The findings that peers felt the compensation they received was incommensurate with their workload and that they often needed to borrow money to achieve their assigned tasks or face threat of economic sanctions from the intervention are similar to findings in research related to CHWs globally [21, 32, 53]. For example, Takasugi and Lee ([40] p.843) noted, ‘As many CHWs are drawn from relatively poor socio-economic groups, they are doubly burdened by poverty and having to carry out unpaid work. It is therefore not surprising that staff retention is an issue.’ Contrary to the view that not increased monetary incentives but context-specific incentives improved CHW retention and the effectiveness and sustainability of programs [37], Bhattacharya et al. [21] found that although monetary incentives could have unwanted repercussions, a satisfactory remuneration, material and/ or financial incentives and possibility of future paid employment were key incentives, and inconsistent remuneration was a disincentive for CHW motivation and retention. Rahman et al. [54] found that monetary factors that influenced retention included the incentives and costs of being a CHW, availability of alternate job opportunities that were more attractive or longer-term than project based work, and the extent to which a CHW’s pre-hire expectations were realized. Similarly, our data indicate that PEs found that at times the costs of being a peer worker outweighed the monetary benefits. Yet they continued to stay on the job because of the expectation that with transition to government funding, their positions would be regularized and provide the security implied in government jobs. Our findings also indicate that the intervention’s de facto practices had economic consequences for peers, which, if unaddressed, could affect their retention and motivation. Intervention practices of collecting in advance the dues for socially marketed condoms, or of not providing full money for rent of facilities, or putting pressure on peers to pay from their pockets for FSW membership in CBOs are examples of such practices. Delayed payment of honorariums, non-availability of travel money for timely outreach work, as well as other decisions such as non-provision of food and drink at peer meetings are other examples of how the intervention’s decisions and practices burdened peers economically. Similarly, CHWs’ reduced motivation has been observed in situations of delay in payment of transport money for outreach work, and change in other incentives like not providing food or drink during day-long meetings [36].

Perhaps ironically, the intervention recognized that FSWs who had dense networks in the FSW community were likely to be the best suited for peer outreach work because such FSWs would be best placed to influence their peers, towards whom the intervention is focused. The intervention’s position acknowledges findings that the effectiveness of CHWs’ work depended almost entirely on their relationship with the community [3, 19, 54]. Once selected, however, the work load of a peer precluded her ability to spend time maintaining her dense sex work connections. If she decreased her time as a peer educator and did not meet her monthly targets, a peer could face various forms of economic sanction, including dismissal. Timely completion of assigned work depended in part on being advanced travel money for outreach work; however, with irregular payments of honorariums and travel money and decreased earnings from sex work, PEs had less money to spend on travel for outreach work. In these ways too, their ability to do the peer work was undermined.

Our findings raise the fundamental question of why the intervention, which recognized the importance of the work done by PEs, did not appear to match that recognition with compensation they deemed adequate. Possible explanations for this situation could not be found in our data, but instead in the literature on CHWs and task shifting. One explanation is that no monetary compensation would ever be satisfactory to all PEs [21]. Yet another explanation appears to be that because peer outreach workers occupied the lowest position in the hierarchy of health personnel in HIV prevention interventions, as part-time semi-literate workers with social rather than technical skills, they had low monetary value for the intervention. Even when paid an honorarium for their work, interventions may consider PEs primarily to be ‘volunteers’ motivated by service to their community, among other reasons. For these reasons, CHWs were found to struggle with securing an ‘economic recognition that is not contained in the discourse of volunteerism’ ([26] p.210). To determine the value of the labor of PEs and a fair compensation for their work, interventions need to consider that in an under-staffed public health care system [55], PEs are the frontline health personnel who reach underserved, stigmatized FSW populations. They also need to consider the benefits of paying fair and regular wages to economically insecure women and the effects to HIV prevention outcomes in not doing so. CHWs are a good investment in HIV prevention but, as Lehmann & Sanders ([32] p.vi) observe, they are ‘neither a cheap option to provide access to health care for underserved populations nor a panacea for weak health systems.’

Our findings must be read with some caveats. We attempted to reduce potential observation and respondent bias through observations of a wide range of meetings and informal conversations with people of different ranks and viewpoints. Although we sampled observations and people from three sites, the sample is small and purposive and could affect the generalizability of findings to other similar interventions. Coding by only one researcher could be yet another bias. Lack of access to data on program budgets and PE retention rates at the study sites limits the depth of this analysis. Despite these limitations, we believe that the findings are likely to be similar at other HIV prevention interventions that work with FSWs as peer outreach workers.

## Conclusion

Peer educators take time out from other livelihood activities to engage in outreach for HIV prevention. Interventions need to match their recognition that PEs add value to HIV prevention efforts with adequate and timely payment of wages and adequate budgetary resources for outreach and community mobilization work. Only by creating conditions which retain, motivate, and support PEs to nurture their relationship with FSW communities are interventions likely to succeed in affecting HIV prevention outcomes.
